# How the Intake of Pulses May Impact Metabolic Disorders and Dementia Risk: A Narrative Review

**DOI:** 10.3390/nu17243898

**Published:** 2025-12-12

**Authors:** Lisa M. B. Salinas, Maricarmen Marroquin, Mariana Mendez, Isabel Omaña-Guzmán, Juan C. Lopez-Alvarenga

**Affiliations:** 1 Department of Nutrition & Dietetics, University of Texas Rio Grande Valley, Edinburg, TX 78539, USA; 2 School of Medicine, University of Texas Rio Grande Valley, Edinburg, TX 78539, USA; 3 Centro Especializado de Diabetes y Metabolismo (CEDIAMET), Universidad Mexico Americana del Norte, Reynosa 88640, Tamaulipas, Mexico; 4 Pediatric Obesity Clinic and Wellness Unit, Hospital General de México “Dr. Eduardo Liceaga”, Mexico City 06720, Mexico; 5 Division of Population Health & Biostatistics, University of Texas Rio Grande Valley, Edinburg, TX 78539, USA; juan.lopezalvarenga@utrgv.edu

**Keywords:** pulses, dementia, Alzheimer’s Disease, diet, metabolic disorders, health

## Abstract

We present a narrative review focusing on pulses’ geographical origin and distribution, their impact on human evolution and history, and their influence on human health. Pulses, including dry peas, beans, and lentils, are renowned for their richness in chemical antioxidants. Despite containing antinutrients, processing techniques preserve their health advantages. Epidemiological research has consistently demonstrated that the consumption of pulses is associated with favorable effects on metabolism. This evidence is further supported by molecular and clinical research, which has elucidated potential nutrigenomic mechanisms and effects on gut microbiota composition underlying their health benefits. However, the literature lacks randomized controlled clinical trials investigating the effects of pulses on health outcomes. Despite this limitation, our review provides valuable insights into the potential beneficial effects of pulses in ameliorating metabolic disorders and reducing the risk of dementia and Alzheimer’s disease. Acknowledging the current limitations, we identify areas for further research to generate additional evidence. Specifically, well-designed randomized controlled trials are needed to thoroughly assess the efficacy of pulses in preventing metabolic diseases. Addressing these research gaps will enhance our understanding of the health benefits associated with pulse consumption and facilitate evidence-based dietary recommendations to improve public health outcomes.

## 1. Introduction

In recent decades, significant strides have been made in enhancing quality of life, with one notable advancement being the Green Revolution, also known as the Third Agricultural Revolution. This transformative movement, initiated in the previous century, stemmed from technology transfer initiatives to bolster crop yields. Initially taking root in developed nations, the Green Revolution swiftly gained global traction, extending its influence until the late 1980s [1]. Concurrently, the emergence of genomic knowledge and biotechnological advancements empowered scientists to manipulate genes, offering new avenues for crop improvement and agricultural sustainability. However, despite these advancements, challenges such as environmental changes threaten food security.

Additionally, demographic modifications to increase the aging population have brought concerns about dementia risk. Social conditions such as undernourishment resulting from food insecurity and the proliferation of fast food contribute to metabolic disorders, exacerbating the risk of vascular dementia and Alzheimer’s disease (AD). Within this context, we focus on an unconventional player in the slow green revolution-beans or pulses (rice and maize are the fastest players). By exploring their genomic makeup, evolutionary significance, and nutritional and cultural relevance, we aim to elucidate the potential beneficial effects of pulses in ameliorating metabolic disorders and mitigating the risk of dementia and AD. Our paper comprehensively explains pulses and their possible implications for AD prevention.

## 2. Legumes and Pulses

Though the words ‘legumes’ and ‘pulses’ are frequently used interchangeably, pulses fall under the umbrella of the legume family. In other words, all pulses are legumes, but not all legumes are pulses. Any plant from the Fabaceae family is called a legume, which includes all plant parts, including the leaves, stems, and pods. Pluses are the dried, edible seeds of specific leguminous plants, including dry peas, beans, and lentils [2].

The legume family appeared on Earth 50 million years ago and includes pulses, soy, green beans, wax beans, and other related plants, like fresh peas and peanuts. The words legume and pulse have Latin roots. The former derives from the verb lĕgo, meaning to collect, seize, extract, and remove. Leguminibus (Latin for legumes) were probably all sorts of grains in pods whose seeds were collected inside them. The word pulse came from the ancient Roman dish puls. The puls is obtained by mixing the flour of some legumes or cereals with hot water, milk (when available), and other ingredients such as wine, pig’s fat and fagots, crushed pepper, and salt to give a final product like an emmer porridge [3]. The legumes cultivated in Greco-Roman areas were chickpeas (*Cicer arietinum* L.), fava beans (*Vicia faba* L.), lentils (*Lens culinaris* Medikus), peas (*Pisum sativum* L.), lupins (*Lupin* spp.), as well as black-eyed peas (*Vigna unguicolata*).

## 3. Genetic and Cultural Evolution of Pulses

The fava bean (*Vicia faba*) genome is a whopping 13 gigabases long [4], about 4 times the size of the human genome, and bigger than most other crop genomes. Otherwise known as the faba bean or the broad bean, the fava bean is a popular protein source in the Middle East, Mediterranean, and South America [5]. The common bean (*Phaseolus vulgaris*) is the most consumed grain legume worldwide. It is adapted to a wide range of climates. Some species of pulses, like *P. acutifolius* (the tepary bean native to Mexico and the US Southwest), have been adapted to dry climates, becoming attractive for their heat and drought tolerance [6]. The common dry bean exhibits tremendous variability (>40,000 varieties) produced in small-scale crops grown in Latin America, Africa, and Asia [7].

The genome of legumes, including pulses, contains repetitive elements known as long terminal repeat (LTR) retrotransposons, which are categorized into 165 families. Some of these elements are ancient, with origins dating back more than 10 million years, while approximately 20% are considered more recent, appearing less than 500,000 years ago. The divergence between a legume known as the common bean (*Phaseolus vulgaris*) and soybean (*Glycine max*) occurred approximately 19 million years ago [8].

The process of domesticating the *Phaseolus* species spans over 10,000 years, resulting in the domestication of five out of approximately 75 known species. Interestingly, ancient Greek texts referred to beans as “fasiolos,” likening their shape to that of a light sailing boat with a bean-shaped hull [9]. Multiple domestication events occurred in regions such as Mexico and the Andes, unlike maize, which underwent a single domestication process. Genetic studies based on five loci suggest that *Phaseolus* originated in Mexico before spreading to the Southern Hemisphere [10]. While domestication reduced genetic variability, hybridization between wild and domesticated varieties also took place. The analysis of chloroplast genomes, which consist of approximately 55 kb, facilitates the examination of group variation.

The Popol Vuh, a sacred text of the Mayan people, identifies fourteen different food plant species, including lima beans (*Phaseolus lunatus*), cocoa (*Theobroma cacao*), tomato (*Solanum lycopersicum*), and chili (*Capsicum annuum*) [11]. In pre-Columbian times, there was limited trade between regions, but significant expansion occurred in the seventh century when colonists introduced beans to Spain, and the Portuguese brought beans to Brazil and East Africa. Beans became a staple food for travelers both at sea and on land.

Globally, over 12 million tons of dry beans are produced annually, with 81% of this production occurring in tropical countries. Brazil is the leading producer and consumer of beans [12].

In 2022, Tridge reported that Britain imported 100.9 metric tons of baked beans, valued at approximately $99.1 million [13]. Other countries like Niger eat an impressive 363 kcal per day of pulses [14], with dried black-eyed peas being one of the more popular types. Black beans are most consumed in Brazil and Guatemala, red-colored beans are the preferred bean in Nicaragua, and white beans are more likely to be consumed in Peru. Additionally, urd beans, mung beans and chickpeas are popular in India and Pakistan, the lentil is most preferred in eastern India, Bangladesh, and Nepal, and, in southern India, consumers often seek pigeon peas above other pulses. It is in China and other East Asian countries where mung beans and adzuki beans are the most consumed, and large, red-colored beans are more likely to be consumed in Columbia and Mexico [15].

Furthermore, beans are commonly and traditionally cultivated in regions like Mexico and Central America, though it is rare to find fields dedicated solely to beans. Often, beans are grown within the same area as maize (corn). To this day, dry beans are typically found in many Hispanic cultures. The potential for incorporating dry beans into American cuisine exists due to the influence of Hispanic culinary styles such as Mexican and Tex-Mex [16].

## 4. Pulse Intake, Knowledge, and Attitudes

According to the US National Health and Nutrition Examination Survey (NHANES) in addition to other smaller surveys surveying the consumption of pulses (beans, lentils, and peas) [17], intake of this food category is low. Less than 5% of study participants reported daily legume consumption. Only about 33% of participants consumed legumes within the last month [17]. Another study examining NHANES data from 2004–2014, found that the average pulse intake in the total population was 20 g/day [18], which equates to approximately ½ cup per week. Other research examining NHANES 2017–2018 data revealed that, of 4741 adults, 20.5% consumed legumes in the past 24 h [19]. Purchases have reflected even lower intake, where the average annual per person expenditure on legumes was $4.76 between 2017–2019 in the US. Those who consumed legumes were more likely to be Hispanic, highly educated, and from a larger household. In a small pilot study of low-income men, 79% consumed beans at least 2–3 times/month [20]. Importantly, the most recent Dietary Guidelines for Americans 2020–2025 (DGA) [21] in the context of a 2000-calorie diet, recommend consuming at least 1.5 cups of pulses or legumes per week, which amounts to 3, ½ cup servings per week.

Pulse intake in older adults may be more promising. In a population of ≥65-year-old, primarily white US adults, 51.2% reported daily or weekly bean consumption. Additionally, 99.2% of this older adult population thought that beans were healthy, 98.0% acknowledged that beans could improve their health, 83.6% stated that beans were high in dietary fiber, and 84.8% were aware that beans could support heart health. However, it was only the bean consumers who were significantly more likely to link bean consumption to fiber-related health issues, like body weight management and constipation, or that beans were low in elements related to heart health, such as total fat, saturated fat, trans fat and cholesterol [22]. Bean consumers were also more likely to report motivating factors related to bean consumption, such as nutritional benefits, taste/texture, and ingredient flexibility. It was these qualities that predicted bean consumption. Bean non-consumers were more likely to report barriers to bean consumption, such as flatulence/abdominal discomfort, lack of knowledge about preparation/cooking, and not perceiving beans as a part of their traditional diet. These barriers predicted the non-consumption of beans [23]. As pulse intake is lacking [17,18], it is important to acknowledge and reduce the barriers preventing consumption. Besides the barriers mentioned above, others include concern about ‘antinutrients,’ unsure preparation knowledge and assumptions of long cooking times, and the idea that pulses are only for low-income populations [24]. Additionally, individuals may perceive pulses as merely suitable as a side dish rather than a central player in a recipe or meal.

Like the US, the average intake of pulses internationally is approximately 21 g per person daily. However, there is considerable variation in intake, depending on global location. The highest average per capita pulse consumption is within Latin America and the Caribbean (34 g/day), sub-Saharan Africa and South Asia (33 g/day). No matter where in the world, pulse consumption has stagnated over the past three decades, and this is likely due to the rise in dependence on animal-origin foods to meet protein needs. Interestingly, there is an inverse relationship between income and pulse consumption in many areas of the world, yet not in India or Tanzania, two of the highest pulse-consuming countries. It is in these locations where middle- and high-income households are more likely to consume pulses than low-income households [15].

In the areas in the world called ‘The Blue Zones,’ where they have the highest percentages of individuals who live over 100 years of age, one of the 9 main common factors they share is a plant-based diet that is remarkably legume/pulse-dominant [25]. For example, in Costa Rica, 80% of the population consumes pulses/legumes daily; in Sardinia, 60% of the population consumes 2–5 servings of pulses/legumes per week [26]. Various dietary patterns from around the world, including the Mediterranean diet, the Dietary Approach to Stop Hypertension (DASH) diet, and the Mediterranean-DASH diet Intervention for Neurodegenerative Delay (MIND) diet [27], as well as diets from Asia, Latin America, and Africa [28] are plant-forward, include pulses/legumes, and have been linked with improved chronic disease risk.

## 5. Nutritional and Antinutritional Properties of Pulses

### 5.1. Processing of Pulses

The two main forms of pulses available in the US marketplace are dry or canned. Each option has its benefits and costs. Firstly, dry pulses require less overall processing and provide greater control over the sodium in a prepared dish. Additionally, the cost per serving of dry beans is less than that of canned beans. One drawback is that dry beans take longer to prepare as they require some soaking before use, depending on the method of soaking and cooking. One way to circumnavigate the soaking issue is through batch cooking [29]. Lastly, dry beans have more iron, magnesium, manganese, potassium, and zinc than canned beans [30]. On the other hand, canned beans contain more sodium and decrease cooking time as they can be used immediately out of their package [29]. Research suggests that canned beans also contain more calcium than dry [30].

Furthermore, research shows that draining and rinsing can eliminate up to 41% of the sodium in canned beans [31]. Interestingly, culture may play a role in eating dry or canned pulses. In a study of a Brazilian population, the majority of consumers refused to consume ‘pre-cooked’ canned beans [32]. Likewise, in a low-income Latina female population in the US, those less acculturated individuals preferred dry to canned pulses [33,34].

### 5.2. Antinutritional Factors

Antinutritional factors (ANFs) in pulses include lectins, oligosaccharides, phytic acid, saponins, and tannins [35]. ANFs can be concerning in pulses, causing harmful effects, especially if pulses are ingested raw, but may impact the bioavailability and digestibility of nutrients and minerals if consumed at high doses, regardless of if cooked or not [35,36,37]. However, food technology has made great strides in processing by incorporating extrusion, microwaving, fortification, fermentation, bioprocessing, etc., alongside more traditional methods, which clears a path toward safe and nutritious human consumption [38,39,40]. Specifically, the oligosaccharides seem to be the culprit for abdominal pain, bloating, and flatulence reported with dietary-fiber-containing foods like pulses [41]. However, there is some evidence that these elements of gastrointestinal discomfort may wane with gradual exposure. In an adult population, where they were fed ½ cup of pinto, navy beans, or black-eyed peas for 8–12 weeks, <50% of the participants reported flatulence during the first week after consuming pinto or navy beans, and only 19% of the participants who consumed black-eyed peas reported increased flatulence. In both groups, flatulence decreased considerably over the next 7 weeks. Bloating, which is sometimes reported with the intake of pulses, was reported in only 29% of participants at the start and showed the same decrease over time as flatulence [42]. Fascinatingly, in small amounts, some ANFs like ɑ-galactosides, bioactive peptides, lectins, oligosaccharides, phytic acid/phytate, protease/amylase inhibitors, and saponins may exert positive bioactive effects in the body [43]. These effects will be discussed further under the subheading ‘Antioxidants’.

## 6. Chemical Constituents of Beans and Their Effects on Human Health

### 6.1. Antioxidants

Pulses contain a variety of compounds that provide antioxidant and anti-inflammatory effects (Table 1). Some compounds, such as carotenoids, dietary fiber, non-starch polysaccharides (NSPs), and phytosterols, solely exert positive effects, while others, such as oligosaccharides (α-galactoside in particular), phytic acid and polyphenols, can act as a bioactive antioxidant or anti-inflammatory compound at low doses, but an antinutrient at high doses [37,43].

In general, when a weight-loss/cholesterol-lowering, pulse-based diet is implemented, lipid peroxidation biomarkers decrease, thereby diminishing overall oxidative stress [44,45]. Incorporation of functional foods made with fiber-containing pulses can decrease glycemic load and reduce C-reactive protein and other markers of inflammation and oxidative stress [46].

Carotenoids are one example of antioxidant compounds found in many pulses. They act as lipid antioxidants, thereby promoting the antioxidant defense of low-density-lipoprotein (LDL) cholesterol in the blood against lipid peroxidation [47,48]. Pulses also contain a significant amount of dietary fiber [36], an average of 5.5 g per ½ cup [49]. Soluble and insoluble fiber are found in pulses and directly and indirectly exert antioxidant effects. Soluble fiber improves glucose homeostasis by binding to glucose in the blood, causing fewer advanced glycation end-products, often leading to decreased acute-phase inflammation markers [45,46]. In addition, the fermentation of dietary fiber in the gut produces short-chain fatty acids (SCFAs), which seem to exert anti-inflammatory action in overweight and obese individuals [50], the critically ill [51] as well as in the general human population [45,52]. Non-starch polysaccharides (NSPs) are found in the cell walls of some beans and pulses [53] and are another group of compounds that are suggested to exert antioxidant effects as well as regulate aspects of immune function [54,55,56,57]. Phytosterols are yet another group of nutrients found in pulses. There is strong evidence that phytosterols help lower LDL cholesterol [58,59]. Tocopherols are found in several forms (ɣ-tocopherol δ-tocopherol) in various pulses [36,48,60,61]. They play an antioxidant role in the body through their ability to prevent lipid peroxidation. They scavenge free radicals and protect membranes and lipid molecules from peroxidation [36].

Oligosaccharides exert beneficial effects at low levels [43]. One specific oligosaccharide is α-galactoside, and while it may cause gas and flatulence in some individuals, at low doses (no more than 3 g/day), it acts as a prebiotic and is fermented in the colon. SCFAs are released upon fermentation, supporting the immune system and decreasing inflammation [62]. Phytic acid is a constituent of beans, grains, and oil seeds. While at higher levels, it may prohibit mineral absorption and overall mineral bioavailability, at low levels, it has significant antioxidant properties [43,63]. Polyphenols are a large group of immune-promoting antioxidants found in plants and pulses [59], which regulate inflammation, oxidative damage, autophagy, and apoptosis pathways [64]. A subgroup of polyphenols highly represented in some pulses is called anthocyanins [36,48]. Overall, polyphenols have been tied to the management of aging-related diseases, including metabolic syndrome, neurodegenerative diseases, and some cancers [64,65,66], as well as hypertension, type 2 diabetes mellitus, skin hyperpigmentation, AD, psoriasis, acne, allergies, cardiac disease and osteoporosis [66]. Nevertheless, some evidence suggests that polyphenols could interfere with nutrient absorption at very high levels [43]. Protein hydrolysates, readily found in pulses [67], while acting as antinutrients at high levels, modulate inflammation and oxidative stress at lower levels. They have been shown to act as free radical scavengers, chelate pro-oxidative transition metals, inactivate reactive oxygen species, and inhibit lipid peroxidation [45,67,68]. Saponins are another example of compounds that may positively and negatively affect health. On one hand, they are known to diminish cholesterol absorption and scavenge free radicals. On the other hand, in very high amounts, they may decrease nutrient absorption [45].

### 6.2. Nutrigenomics of Pulses and Legumes

Nutrigenomics analyzes the effect that nutrients have on the genome [69]. Evidence about the nutrigenomic effects of biochemical compounds of pulses is scarce. The compounds that have been most studied are those found in black beans (BB), primarily anthocyanins.

The beneficial effects of anthocyanins could result from epigenetic modifications that regulate the expression of genes involved in different inflammatory and cardiometabolic alterations. However, the specific mechanisms need to be studied better [70]. In a study conducted in an animal model to analyze the anti-diabetic properties of an extract rich in anthocyanins from BB on adipose tissue, it was observed that BB extract could modulate the expression of genes involved in the pathogenesis of type 2 diabetes mellitus (e.g., insulin secretion and phosphatidylserine binding). Furthermore, this study suggests that anthocyanin modulates non-coding RNAs, such as miRNAs and lncRNAs. Globally, it was observed that the genes whose expression is modulated by BB are related to metabolic and cardiovascular diseases, as well as nutrition disorders and immune system diseases [71]. Another study in vitro observed that the cyanidin3-glucoside, an anthocyanin derived from BB, increased the expression of GLUT4 in adipocytes [72].

The effect of BB flavonoids and saponins on lipid metabolism has also been studied in vitro and in an animal model [73]. In this study, it was observed that flavonoids and saponins reduced the expression of SREBP1c (sterol regulatory element binding protein-1c), FAS (fatty acid synthase), and HMGCR (3-hydroxy-3-methylglutaryl-CoA reductase) proteins in hepatocytes. SREBP1c is a transcription factor that induces the expression of a family of genes related to glucose utilization and lipogenesis. FAS is an enzyme needed in the lipogenesis pathway [74] and HMGCR is an important enzyme in cholesterol synthesis [75]. Furthermore, flavonoids and saponins from BB increased the expression of the ABCG5/ABCG8 and CYP7A1, which are transporter proteins in the reverse cholesterol transport [73].

The anti-cancer activity of beans has been studied in an in vitro study [76] that evaluated the effect of black turtle bean extract on breast cancer cell lines, finding an increase in the expression of the Bax gene and a decrease in the expression of the Bcl-2 and Bcl-xL genes. Bax is a proapoptotic protein, while Bcl-2 and Bcl-xL are antiapoptotic proteins [77]. Additionally, the activity of the enzymes caspase-3/7 that participate in the apoptosis pathway was increased.

The BB protein has also been shown to affect lipid metabolism by modulating gene expression. A study in an animal model [78] observed a decrease in the expression of SREBP-1 and FAS, an increase in PPARα and CPT-1, and a reduction in glucose and insulin.

Research regarding nutrigenomics and pulses is limited. However, some exciting evidence is investigating the effects of the anthocyanins found in Korean black soybeans (KBSB) (part of the broader legume category) on nutrigenomic elements. In general, it has been observed that anthocyanins from legumes have a neuroprotective effect in animal models through several mechanisms [79,80]. The effect of anthocyanins extract from KBSB on the hippocampus and cortex regions was evaluated in an induced oxidative and inflammatory animal model [80]. In this study, the anthocyanin extract decreased the expression of the receptor for advanced glycation end products (RAGE) that had a role in the inflammatory response and acted as a receptor of amyloid-β (Aβ) peptides. Aβ peptides participate in the development of Alzheimer’s disease, and their binding with RAGE allows their entry from the circulation into the brain [81]. Additionally, the union of these molecules activates the nuclear factor kappa-light-chain-enhancer of activated B cells (NF-κB) signaling pathway involved in inflammation [81]. Consequently, in the study, the anthocyanin extract showed an improvement in the behavioral performance and a suppression of inflammatory markers such as phospho-NF-κB (p-NF-KB), inducible nitric oxide synthase (iNOS), and tumor necrosis factor (TNF-α) as well as in astrocytes and microglia cells, which also participate in the pathogenesis of Alzheimer’s disease.

Another study [79] estimated the effect of the same anthocyanin extract from KBSB on neurodegeneration through antioxidant mechanisms. The administration of anthocyanins upregulated the expression of p-PI3K, p-Akt, p-GSK3β (Ser9), and Nrf2 involved in antioxidant pathways. The expression of target genes (HO-1 and GCLM) of Nrf2 was also increased. The authors concluded that anthocyanins had neuroprotective effects in Alzheimer’s mouse models by regulating p-PI3K, p-Akt, p-GSK3β pathways, and Nrf2/HO-1.

Though the two studies investigating KBSB are reflective of the effects of legumes, anthocyanins are also found in significant amounts in pulses, as evidenced by the studies outlined at the start of this section [70,71,72].

The findings of studies about the nutrigenomic effects of flavonoids, anthocyanins, saponins, and proteins in beans have been promising in preventing and treating cardiometabolic and neurodegenerative diseases. However, these studies need to be translated to humans and evaluated to determine if similar results are found. Additionally, it is crucial to consider that the extracts of the evaluated compounds are present in much higher concentrations than those found naturally in pulses. Therefore, it is also important to determine what quantity of pulses is associated with the observed beneficial effects in the studies to provide consumption recommendations at the population level.

There is a lack of studies about pulses. It would be interesting to study whether the compounds in lentils and dry peas have similar effects to those in beans. In addition, nutrigenetic studies are needed to investigate whether individual genetic variability influences the observed effects of pulses.

## 7. Microbiome and Pulses

In the last decade, significant research has been carried out on the relationship between the gut and the brain, discovering the existence of a bidirectional communication system between them, thus establishing the term “microbiota-gut-brain axis,” which involves metabolic, endocrine, neural, and immune pathways that are crucial for the maintenance of brain homeostasis [46].

The literature states that the early cognitive impairment phase of AD is characterized by significant alterations of the intestinal microbiome, also known as intestinal dysbiosis [82].

In dysbiosis, the concentrations of essential molecules such as γ-aminobutyric acid (GABA), serotonin, SCFAs, and other molecules necessary for the proper functioning of the central nervous system may be altered, which could lead to the appearance of diseases such as anxiety, AD or autism [83].

**Figure 1:**
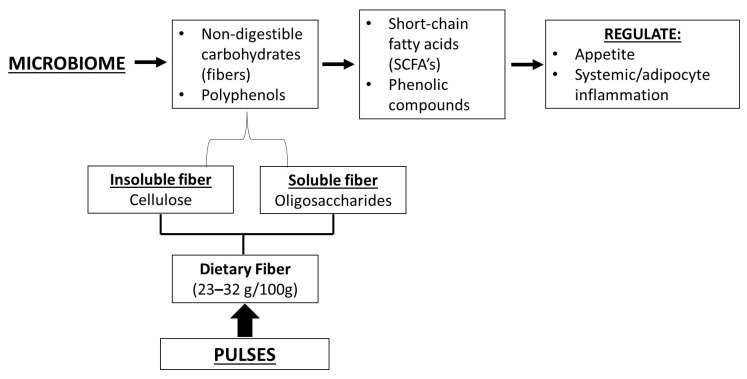
Participation of pulses in the production of short-chain fatty acids (SCFAs) and polyphenols.

Today, we accept that the microbiome acquires these molecules from the fermentation of non-digestible carbohydrates and polyphenols, producing SCFAs and phenolic compounds, which regulate systemic and adipose cell inflammation (Figure 1).

Common beans contain between 23 to 32 g/100 g of dietary fiber, both soluble and insoluble [84], and this fiber content has generated significant interest. Some studies consider the starch derived from legumes a potential prebiotic that increases the abundance of beneficial bacteria in the microbiome.

Bacteria like *Akkermansia muciniphila* and *Prevotella* produce SCFAs (Table 2), are negatively associated with overweight or obesity, and may regulate inflammation [84].

Human and mouse studies have been conducted on microbiome-related bacteria to measure their prebiotic capacity using fiber-rich foods. A study examined the prebiotic effects of legume resistant starch on the intestinal metabolome of 60-week-old mice inoculated with the human microbiome. The mice were fed a Western control diet enriched with resistant starch from pinto beans, black beans, lentils, chickpeas, or inulin as a reference [85]. It was observed that butyrate shows a strong positive correlation with lentils and chickpeas. Propionate shows a weaker positive association with lentils and chickpeas and a negative association with pinto beans and black beans. However, butyrate in lentils is significantly more abundant (*p* < 0.01) than inulin.

Some studies in mice have demonstrated the ability of common beans to modify alpha diversity during a period of obesity. In one such study, where different varieties of common beans were added to a high-fat diet, *A. muciniphila* bacteria increased 2 to 473-fold over 17 weeks. It has also been reported that bacteria typically less commonly found in obesity, such as *Prevotella*, are found more frequently upon consumption of legumes. In a study of 4-week-old male mice supplemented with white beans for 12 weeks, the abundance of *Prevotella* increased 332-fold compared to the control group, and the fecal abundance of SCFAs increased [86].

As for human studies, few studies have been conducted to investigate legume consumption’s effect on individuals’ microbiome, and no conclusive results have been observed, unlike in animal studies. In one study, 80 individuals with metabolic syndrome were fed 130 g/day of pinto beans for 12 weeks; only a decreased abundance of *E. limosum* (a bacterium that produces SCFAs) was observed with bean consumption. They then took fecal samples from these individuals and performed an in vitro fermentation experiment by feeding the microbiota with dried bean powder, corn starch, inulin, and oat bran. The results showed that total SCFAs and propionate increased significantly only when dry bean powder was used as the substrate [87].

Similarly, a pilot randomized controlled clinical trial was conducted on 29 overweight and obese colorectal cancer survivors. They were fed 35 g/day of navy bean powder, 30 g/day of rice bran, or macronutrient-matched control. The supplementation with navy bean powder or rice bran increased intestinal bacterial diversity at 28 days compared to baseline, however only rice bran intake led to a decrease in the Firmicutes: Bacteroidetes ratio and an increase in SCFAs (propionate and acetate) in the feces after 14 days but not at 28 days [88].

Lastly, a randomized crossover dietary intervention trial was carried out in obese patients with a history of precancerous colorectal polyps and colorectal neoplasia. For eight weeks, 55 patients were provided with one cup of navy beans on top of their usual diet. The researchers found an increased diversity and shift in various bacteria, which suggested the beans served as efficacious prebiotics. Additionally, nutrient and microbiome-derived metabolites shifted, and modifications were observed in specific biomarkers related to intestinal and systemic inflammatory response [89].

Although there have not been an adequate number of studies in humans to establish the prebiotic capacity of pulses, most studies in mice have demonstrated their ability to alter the alpha diversity of the microbiota, including increasing the abundance of SCFA-producing bacteria such as *A. municiphila* and *Prevotella*, among others. Pulses may be an ally to prevent systemic inflammation and the production of brain-protective molecules, which may help prevent or delay cognitive impairment. More human research is warranted to establish the effects of pulses on short and long-term markers of systemic inflammation.

## 8. Can Pulses Help Prevent Dementia and Other Cognitive Disorders?

### 8.1. Metabolic Benefits

**Figure 2:**
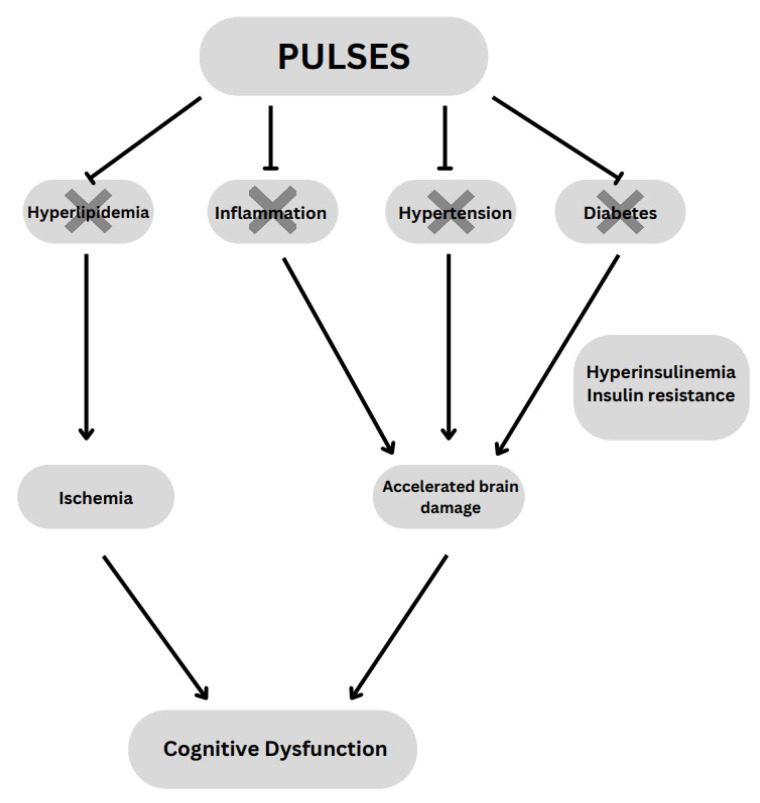
Proposed impact of pulses on the prevention of cognitive dysfunction in the presence of metabolic risk. The “X” mark indicates a protective effect by attenuation of hyperlipidemia, inflammation, hypertension and diabetes due to pulse consumption.

There are several types of dementia, from Lewy-body dementia to AD, and these diseases have differentiated risk factors that can change drastically depending on the disease type. The type of dementia that has modifiable factors that contribute to its disease process is called Multi-Infarct Dementia (MID), and it is a type of dementia that has only been accepted into the medical community within the last four decades. It is considered the second most common reason for dementia, after AD. Risk factors for MID include hypertension, hyperlipidemia, smoking, heart disease, diabetes, and the presence of both inflammation and oxidative stress [90] (Figure 2).

In a study of over 100 MID patients and age-matched controls, researchers found that the G allele of the –174 G/C polymorphism in the IL-6 gene was nine times more likely to occur in the MID group [91]. Pulses are a promising food group that lowers risk factors that contribute to MID. Pulses have been implicated as part of a healthy diet that can reduce weight and glycemic index, decrease diabetes incidence, lower cardiovascular disease risk and mortality, and lower inflammation [92].

Of the factors affecting MID, those most likely to be a significant part of ischemic events are hyperlipidemia and hypertension. Pulses are known for their ability to reduce LDL, the cholesterol subtype most responsible for arterial disease. One systematic review found a correlation between intake of pulses and a moderate decrease in LDL [93] while another systematic review and meta-analysis linked pulse consumption with significantly lower LDL [94]. Dietary pulses may reduce hypercholesterolemia as well as hypertension. Another systematic review and meta-analysis study found that pulse consumption was associated with significantly decreased systolic (Mean differences (MD) = −2.25 mm Hg (95% CI, −4.22 to −0.28), *p* = 0.03) and mean arterial blood pressure (BP) (MD = −0.75 mm Hg (95% CI, −1.44 to −0.06), *p* = 0.03) [95].

When considering vascular disease, it is essential to consider diabetes as a possible risk factor. Diabetes commonly hyalinizes blood vessels, wreaking havoc on the body’s vascular system. That said, diabetes plays a much more insidious part of dementia than simply causing cerebral vascular events. Diabetes has been tied to accelerated damage to the brain, not entirely mediated by microvascular injury [96], but also due to insulin’s role in memory and other cognitive processes. Insulin directly improves declarative memory performance, and insulin receptors are expressed in the hippocampus’s dentate gyrus and CA1 area. Type 2 diabetes causes a disruption of this process by peripheral hyperinsulinemia, reducing the insulin transport into the brain. There is also evidence of a reduction in brain volume in the frontal and temporal lobes among those with chronic hyperinsulinemia [97].

### 8.2. Effect of Pulses on Cognition

As previously mentioned, pulses contain many compounds that positively and independently impact oxidative stress and inflammation [98,99] and both metabolic states are significantly related to the risk of numerous chronic diseases, such as cancer, cardiovascular disease, diabetes and neurogenerative diseases [100]. Evidence is slim, however, about showing a direct impact of pulses on cognition unless this food category is mentioned as a component of a broader dietary pattern, such as the Mediterranean diet, the DASH diet, or the MIND diet [101]. There is a small amount of evidence that dietary patterns that include pulses, alongside fish or seafood, fruits, unsaturated vegetable oils, nuts, and other vegetables support a lower risk of age-related cognitive impairment and/or dementia [101]. Plant protein, compared to animal protein, has been associated with lower odds of subjective cognitive decline [102]. Food categories contributing to plant protein include nuts, beans/legumes, bread, and breakfast cereal. Overall protein intake from animal, eggs, and legume sources positively relates to cognitive function [103]. There has been some modest, regional-specific evidence linking pulse-specific intake to cognition. Bangladeshi adolescent girls were provided lentils, and cognitive status was assessed. There seemed to be a small but significant relationship between lentil consumption and cognition [104]. In a group of Australians aged 70–90 years, higher consumption of legumes and nuts resulted in better performance on a scale of global cognition, as well as in cognitive domains of language and visuospatial [105]. It is important to recognize that, in the study mentioned above, legumes and nuts were combined as one food category, so it is uncertain whether one subcategory was driving the results for the food group as a whole.

## 9. Practical Applications and Future Pulses Research

Considering the low intake of pulses that still exists in the US (½ cup per week or less) [18], even a small increase toward the DGA recommendations of 1 ½ cups per week [21] may improve diet quality, foster meal satiety [106] and provide significantly more health-promoting compounds, thereby supporting chronic disease risk prevention. According to an analysis of NHANES data, only about 7.4% of US adults met the adequate intake for fiber, 14 g per 1000 kcal [107]. Incorporating 1 ½–3 cups of pulses per week will increase the average daily fiber intake by 2.5–5 g of fiber/day [49], allowing consumers to approach the recommended fiber intake. Besides fiber, higher pulse consumption will also contribute numerous other compounds that support health, especially those that function in antioxidants or anti-inflammatory roles.

Enough evidence exists to continue exploring the impact of pulse intake on diet quality, the barriers to pulse consumption, and the prospective impact of increased pulse intake in various populations. Recently, a research group used data from NHANES 2001–2018 and found a correlation between regular intake of pulses (about 6–7 cups per week) and higher intake of several shortfall nutrients, lower consumption of added sugar, healthier BMI, body weight and waist circumference and better diet quality, as measured by the Healthy Eating Index-2015 [108,109]. Exploring the relationship between the consumption of pulses and other measures of healthy dietary intake would add to the small body of work establishing the contribution of pulses to diet quality. The Alternative Health Eating Index (AHEI)-2010 is a measurement of diet quality that focuses on nutrients related to chronic disease risk [110], thus establishing a relationship of this assessment tool with pulse intake would help strengthen dietary guidance regarding the incorporation of pulses into a dietary pattern that supports chronic disease prevention. It is essential to determine what prevents various populations from consuming more nutrient-packed pulses as a regular part of their dietary intake. Does acculturation, where individuals are migrating from traditional eating patterns to Westernized patterns, play a role? It could also be that a stressful lifestyle, poor attitudes towards beans, or perceived gastrointestinal discomfort could also prevent regular intake of pulses, and all justify an investigation. Another barrier might be the lack of cooking confidence when incorporating pulses into a meal or dish. Therefore, culinary nutrition education interventions may be warranted. Dietitian-led culinary nutrition classes could be provided, where individuals learn to incorporate pulses into inexpensive, appealing dishes. Lastly, recent work from the BE-GONE trial demonstrated gut microbiome enhancement after 8 weeks when obese individuals with a history of pre-cancer or colorectal cancer incorporated 1 cup of beans into their daily diet [89].

Future studies should continue the exploration of the impact of pulse consumption on health and disease prevention. Well-designed randomized clinical feeding trials will provide gold-standard evaluation of metabolic outcomes when measured alongside dietary plans containing pulses. They may point to the minimal amount of pulses required to have an effect on short and long-term health, as this has not been consistently quantified, especially in regard to brain health and cognitive function. Research should focus on the gut-brain axis, develop methodology based on hypotheses generated by mechanistic and animal model findings and acknowledge the possibility that ideal dietary patterns may differ among different human subgroups. For example, it will be valuable to determine if factors such as age, sex, chronic disease, race, ethnicity, food security status, and others influence the effect of pulses on health. Forthcoming research must also acknowledge a wide range of barriers that arise with the encouragement of a higher intake of pulses, such as reported GI discomfort, acculturation, and lack of culinary efficacy, and incorporate hands-on nutrition education that supports reducing those barriers.

In summary, pulses are highly nutritious and offer numerous metabolic benefits. With a low glycemic index, pulses help improve glycemic control in individuals with diabetes and may even reduce the risk of developing type 2 diabetes. Their regular consumption is associated with reduced LDL cholesterol levels, lowering the risk of cardiovascular diseases. Pulses are rich in protein and fiber, enhancing satiety and aiding in weight management. Additionally, they contribute to modest reductions in blood pressure due to their high potassium and low sodium content and other beneficial compounds. Pulses also contain antioxidants and anti-inflammatory compounds that help reduce chronic inflammation, a risk factor for many metabolic disorders. Rich in vital nutrients such as iron, folate, magnesium, zinc, and potassium, pulses are an excellent addition to the diet for overall health and metabolic function.

## 10. Conclusions

We reviewed epidemiological and molecular research on pulses’ beneficial effects on cardiometabolic and neurocognitive disorders. Among the potential biological mechanisms identified that could explain these effects are modifications ranging from epigenetic changes to changes in intestinal microbiota. However, there is a lack of randomized clinical trials to provide high-quality causal evidence regarding the consumption of beans or pulses and their potential role in cognitive health prevention. We addressed this gap by presenting findings from laboratory experiments and observational studies, providing valuable insights into the potential health benefits of bean consumption. Supplemental Table S1 presents a summary table of key studies that provide the foundation for further exploring the impact of pulses on metabolic health.

Acknowledging the limitations of the current literature, we identify areas for further research where additional evidence is needed. Specifically, well-designed randomized controlled trials are necessary to assess the efficacy of pulses in preventing and treating metabolic and neurodegenerative diseases. Furthermore, research about the beneficial effects of pulses other than beans, as well as nutrigenetic studies, represents an unexplored research field. By addressing these research gaps, we can enhance our understanding of the potential health benefits associated with bean consumption and inform evidence-based dietary recommendations.

## Figures and Tables

**Table 1:** Physiological effects of antioxidant compounds in dry beans and pulses.

	Physiological Effect	
Compound	Bioactive	Antinutrient
Oligosaccharides	Antioxidant/Prebiotic	Gastrointestinal discomfort
Phytic acid/Phytate	Antioxidant	Interferes with mineral bioavailability
Polyphenols	Antioxidant/Prebiotic	Interferes with nutrient absorption
Protein hydrolysates	Anti-inflammatory	Interferes with protein digestibility
Saponins	Hypercholesterolemic/Antioxidant	Interferes with nutrient absorption

Adapted from: Health Implications and Nutrition Bioavailability of Bioactive Compounds in Dry Beans and Other Pulses [43].

**Table 2:** Bacteria decreased in the alpha diversity (α-diversity) of overweight or obese subjects.

Bacteria in Overweight/Obese Subjects	Metabolites/Functions
Lactobacillus paracasei	Antimicrobial peptides
Lactobacillus plantarum	Antimicrobial peptides
Prevotella	Short-chain fatty acids
Ruminococcus flavefaciens	Degradation and fermentation of resistant starches
Ruminococcus gnavus	Degradation and fermentation of resistant starches
Akkermansia muciniphila	Short chain fatty acids Reduces carbohydrate absorption in jejunum Reduces intestinal barrier permeability May reduce body mass index May reduce waist-hip ratio
Proteobacterias: Escherichia coli and Salmonella	Pathogenic bacteria
Actinobacteria	Pathogenic bacteria

## Data Availability

No new data were created or analyzed in this study. Data sharing is not applicable to this article.

## Supplementary Materials

The following supporting information can be downloaded at: https://www.mdpi.com/article/10.3390/nu17243898/s1, Table S1: Key studies supporting the concepts discussed in this narrative review.

## Abbreviations

The following abbreviations are used in this manuscript:

AD	Alzheimer’s Disease
LTR	Long Terminal Repeat
NHANES	National Health and Nutrition Examination Survey
DGA	Dietary Guidelines for Americans
DASH	Dietary Approach to Stop Hypertension
MIND	Mediterranean-DASH diet Intervention for Neurodegenerative Delay
ANF	Antinutritional Factor
NSP	Non-Starch Polysaccharide
LDL	Low-Density Lipoprotein
SCFA	Short-chain Fatty Acid
BB	Black Beans
SREBP1c	Sterol Regulatory Element Binding Protein-1c
FAS	Fatty Acid Synthase
HMGCR	3-hydroxy-3-methylglutaryl-CoA reductase
KBSB	Korean Black Soybeans
RAGE	Receptor for Advanced Glycation End products
Aβ	Amyloid-β
NF-κB	Nuclear Factor kappa-light-chain-enhancer of activated B cells
p-NF-κB	Phospho-NF-κB
GABA	γ-aminobutyric acid
MID	Multi-Infarct Dementia
MD	Mean Differences
BP	Blood Pressure
AHEI	Alternative Healthy Eating Index
