# Supplementary material for: How the Intake of Pulses May Impact Metabolic Disorders and Dementia Risk: A Narrative Review
Source: Nutrients. 2025 Dec 12;17(24):3898. doi: 10.3390/nu17243898 (PMC12736144; doi:10.3390/nu17243898)
Supplement: Supplementary file 1 [file nutrients-17-03898-s001.zip › nutrients-4015184-supplementary.pdf]

Supplementary Table S1. Key studies supporting the concepts discussed in this narrative review.

| Ref # | Study | Design of the study | Focus | Key relevant findings |
|---|---|---|---|---|
| 18 | Mitchell et al., 2021 | Cross-sectional analysis of NHANES 2003–2014 | Pulse intake from 24-h recalls | Pulse consumers had higher nutrient density and better diet quality, illustrating that even modest pulse intake improves overall dietary profile. |
| 19 | Semba et al., 2021 | Cross-sectional (NHANES 2017–2018) | Legume consumption and grocery purchases | Legumes are inexpensive, but only ~20% of adults reported consuming legumes in the last 24 hr; legumes remain under-consumed despite dietary recommendations. Those who consume them are Hispanic and have a higher level of education. |
| 22, 23 | Doma et al., 2019 | Questionnaire survey | Bean consumption and knowledge | Although most older adults view beans as healthy, only ~51% consume them regularly. There is a behavior-attitude gap relevant to public health messaging.<br>The main barriers in non-consumers are flatulence discomfort and a lack of knowledge in preparation. |
| 43 | Wiesinger & Marsolais, 2022 | Narrative/mechanistic chapter | Bioactive compounds (polyphenols, oligosaccharides, phytate, lectins, etc.) | Describes how multiple bean bioactive compounds act on oxidative stress, inflammation, and nutrient handling. |

| | | | | |
|---|---|---|---|---|
| 5 | Mustafa et al., 2022 | Narrative/mechanistic review | Polyphenols, phytosterols, and non-digestible carbohydrates affect lipids | Summarizes how pulse (specifically lentils) bioactive compounds modulate lipids, glycemia, and gut function, supporting our focus on cardiometabolic and microbiome-mediated benefits. |
| 87 | Finley et al., 2007 | Randomized crossover feeding trial | 130 g/day cooked pinto beans vs control entrée for 12 weeks | Daily pinto bean intake improved serum lipids and altered short-chain fatty acids (SCFA) profiles. The consumption of beans simultaneously acts on microbiota and cardiometabolic risk. |
| 94 | Ha et al., 2014 | Systematic review & meta-analysis of RCTs | Pulses ≈130 g/day (about 1 serving) | Pooled pulse intake lowered LDL-C by ~0.17 mmol/L, providing quantitative evidence for the lipid-lowering effects of routine pulse consumption. |
| 95 | Jayalath et al., 2014 | Systematic review & meta-analysis of controlled feeding trials | Dietary pulses as part of controlled diets | Dietary pulses significantly reduce blood pressure in people with and without hypertension, reinforcing their role in reducing vascular risk. |
| 81 | Kim et al., 2016 | Systematic review & meta-analysis | Pulses incorporated into energy-restricted or weight-maintenance diets | Pulse consumption produced modest but significant weight loss even without strict energy restrictions, supporting our discussion of body-weight regulation. |
| 85 | Kadyan et al., 2023 | Experimental study and mechanistic synthesis | Resistant starch from pulses | Pulse-derived resistant starch promotes beneficial gut microbes and SCFA production, providing a mechanistic link between pulses, microbiota, and healthy ageing. |
